# Sonoporation with Echogenic Liposomes: The Evaluation of Glioblastoma Applicability Using In Vivo Xenograft Models

**DOI:** 10.3390/pharmaceutics17040509

**Published:** 2025-04-11

**Authors:** Ju-Hyun Park, Yoo-Kyung Lee, Hana Lee, Dong-Hyun Choi, Ki-Jong Rhee, Han Sung Kim, Jong-Bum Seo

**Affiliations:** 1 Department of Biomedical Engineering, Yonsei University, Wonju 26493, Republic of Korea; 2 Department of Biomedical Laboratory Science, Yonsei University, Wonju 26493, Republic of Korea

**Keywords:** echogenic liposomes, sonoporation with microbubbles, ultrasound contrast agent, drug carrier for sonoporation, glioblastoma

## Abstract

**Objective**: In previous studies, echogenic liposomes with liquid and gas cores were analyzed as alternative carriers of drug molecules and cavitation nuclei for sonoporation. The possibility of small interfering RNA (si-RNA) encapsulation has also been presented. In this study, the usability of echogenic liposomes as drug carriers and cavitation seeds was evaluated using an in vivo model. **Methods**: A doxorubicin-loaded echogenic liposome was synthesized as a drug carrier. The size distribution and the number of formed echogenic liposomes were measured. Five comparative in vivo experiments were conducted with and without doxorubicin-loaded echogenic liposomes, and the results were statically analyzed. **Results**: Sonoporation with doxorubicin-loaded echogenic liposomes at 3.05 W/cm^2^ of ISPTA ultrasound sonication and 0.98 MHz results in an average tumor volume growth of less than 25% of that following the simple administration of doxorubicin. Considering the *p*-value between the two groups is approximately 0.03, doxorubicin-loaded echogenic liposomes were effectively applicable as cavitation nuclei for sonoporation. **Conclusions**: Although further studies are needed to clarify the responses to incident ultrasound fields, the proposed echogenic liposome appears to be a promising alternative cavitation nuclei/carrier for sonoporation.

## 1. Introduction

The main mechanism of sonoporation, which uses ultrasound to mediate drug delivery to target cells or organs, is cavitation in the vicinity of targets [1,2]. Microbubbles oscillate according to pressure changes under low-amplitude ultrasonic field, which is called stable cavitation. If stable cavitation occurs near the physical barrier, microstreaming is induced due to the non-symmetric boundary condition, and shear stress is exerted on the barrier [3,4,5,6]. On the other hand, if microbubbles are subject to a higher amplitude ultrasound, they rapidly grow and subsequently collapse, a phenomenon that is called inertia cavitation. If inertia cavitation occurs, transit channels can be formed on a lipid bilayer membrane [7,8,9,10]. In either case, sonoporation can effectively deliver drug molecules into spatially targeted cells [10,11,12,13]. Because sonoporation is only effective in the immediate vicinity of the cavitation site [14,15], appropriate cavitation nuclei are central factors in sonoporation.

The main mechanism of sonoporation, which uses ultrasound to mediate drug delivery to target cells or organs, is cavitation in the vicinity of the target [1,2]. Stable cavitation can induce microstreaming and transiently perturb the permeability of the cell membrane [3,4,5,6,7,8], while inertia cavitation can create transit channels on a lipid bilayer membrane [9,10,11,12]. In either case, sonoporation can effectively deliver drug molecules into spatially targeted cells [12,13,14,15]. Because sonoporation is only effective in the immediate vicinity of the cavitation site [16,17], appropriate cavitation nuclei are central factors in sonoporation.

Engineered microbubbles known as ultrasound contrast agents (UCAs) typically have inert gas cores and are stabilized with biocompatible shells [18,19]. When injected intravenously, UCAs can safely enhance contrast in ultrasound imaging [18,20,21,22,23]. UCAs and their derivatives have also been researched as cavitation nuclei for sonoporation [24,25,26,27,28]. Among these derivatives, microbubble- or droplet-conjugated liposome complexes and nested microbubbles or droplets provide a three-dimensional (3D) space for drug uploading and ensure high concentrations of drug molecules at the cavitation site.

In order to load drug molecules, researchers have proposed encapsulating microbubbles or droplets in a liposome-like complex or conjugating a drug-loaded liposome with the shell of the microbubble/droplet [27,28,29,30]. Creating these complex structures typically requires multiple steps, beginning with the formation of the microbubble/droplet, followed by conjugation and/or encapsulation. When creating these complexes from a microbubble, many microbubbles degenerate and change their characteristics, such as size distribution, during the second step due to their limited lifetime at atmospheric pressure [31]. Even though Ibsen et al. reported on the possibility of uploading a payload with the nested microbubbles formed by a multi-step process [27], the usability of the proposed nested microbubbles for cancer treatment has not yet been reported.

In a previous study, a single-step procedure was carried out to create echogenic liposomes, also known as nested microbubbles [32], using a simple overdose of liquid perfluorobutane, which has a boiling temperature of approximately −2 °C, during bubble formation. If the formed microbubbles are then cooled in a refrigerator to approximately 3 °C, some of the excess perfluorobutane appears to recondense due to the high pressure and low temperature. The majority of the formed microbubbles are nested within an internal liquid that provides a 3D space for drug upload and can be termed echogenic liposomes. A small portion of small interfering RNA (si-RNA) can be safely encapsulated and protected from the effects of external RNAse. Unfortunately, the exact measure of payload at the formation stage cannot be provided due to the limited lifetime of echogenic liposomes when they are exposed to the atmosphere. To measure the payload, echogenic liposomes need to be isolated from the base solution. However, their half-lives are limited by several minutes based on the brightness change in ultrasound images, of which their target was echogenic liposomes in degassed water. Therefore, a larger amount of drug-loaded echogenic liposomes would be dissolved during the preparation procedure. This hinders the validation of the usability of echogenic liposomes.

To advance the exact measurement of payloads, which would require a complex experimental setup, in vivo experiments targeting glioblastomas to verify the usability of echogenic liposomes as drug-loadable cavitation seeds are described in this study. Glioblastomas were selected as target cells because attempts to use sonoporation with cavitation seeds to deliver drugs across the blood–brain barrier (BBB) have yielded promising results [33,34,35], and glioblastomas are common primary brain tumors [36,37]. The results were statistically analyzed and discussed in the following sections.

## 2. Materials and Methods

### 2.1. Synthesis of Echogenic Liposomes and Encapsulation of Doxorubicin

The base solution was prepared by diluting deionized water, glycerol (Sigma Aldrich, St Louis, MO, USA), and propylene glycol (Acros Organics, Geel, Belgium) in a ratio of 20:1:21 by volume. In 100 mL of the lipid saline solution, 0.1 g of 1,2-dipalmitoyl-sn-glycero-3-phosphocholine (DPPC) (Avanti Polar Lipids Inc., Alabaster, AL, USA), 0.01 g of 1,2-dipalmitoyl-sn-glycero-3-phosphate (DPPA) (Avanti Polar Lipids Inc.), and 0.03 g of cholesterol (Avanti Polar Lipids Inc.) were added. The solution was heated at 60 °C for 30 min to dissolve the DPPC, DPPA, and cholesterol powders (PC-420D; Corning Inc., Corning, NY, USA). After complete dissolution, 2 mL of the solution was transferred to a vial and cooled for at least 1 h in a refrigerator at −15 °C. Next, 20 µL of liquid-phase perfluorobutane was added, and the prepared solution was high-shear mixed for 45 s using a Vialmix apparatus (Lantheus Medical Imaging Inc., North Billerica, MA, USA). This formed echogenic liposomes with gas-phase perfluorobutane generation under overpressure (approximately 3 psi) inside a 2 mL vial as the temperature of the vial reached 40 °C immediately after vial mixing [14]. The mixed solution was cooled at 3 °C at least for 1 h.

A 2 M doxorubicin solution was prepared for in vivo experiments (12.4 mM) by dissolving doxorubicin hydrochloride (Sigma) in deionized water. Next, 10 µL of the prepared doxorubicin solution and 20 µL of perfluorobutane were added to 2 mL of the prepared solution of DPPC, DPPA, and cholesterol. The mixture was high-shear mixed for 45 s using a Vialmix. The mixed solution was cooled at 3 °C for at least 1 h.

### 2.2. Evaluation Population Characteristics of Formed Echogenic Liposomes

The limit of adopting the high-shear mixing method to synthesize echogenic liposomes is the impurity of the formed bubbles. As shown in a previous report [38], approximately 70% of formed bubbles could be visually confirmed as echogenic liposomes, while the remaining 30% were mostly simple bubbles with occasional simple liposomes. The difficulty of isolating echogenic liposomes is related to the relatively short half-life of around ten minutes in the atmosphere. In order to validate changes in bubble population after centrifugation, which is widely used as a sorting method of bubbles, the total population change was quantitatively measured after centrifugation was conducted NanoSight NS300 (Malvern Panalytical, Malvern, UK). One milliliter of the synthesized echogenic solution was extracted after one-minute depressurization, as mentioned in the methodology. The solution was transported in microtube and centrifuged at 300 RCF (Model 1730R, LaboGene, Seoul, Republic of Korea) for ten minutes. Then, the solution was mixed again with gentle pipetting for ten times. Identical experiments were conducted four times for the purpose of measuring the statistics.

### 2.3. Confirmation of Drug Load Ability Through Electron Microscopy

Gold nanoparticles with a diameter of 15 nm were added during the synthesis of echogenic liposomes. The structure of the echogenic liposome was analyzed using energy filtering TEM (EF-TEM). The produced echogenic liposome was diluted at a ratio of 1:10 in distilled water. Ten microliters of the diluted solution were placed on a 200 mesh Cu TEM grid (Ted Pella Inc., Redding, CA, USA). Negative staining was applied using uranyl acetate. The stain residue was removed by washing twice in distilled water for five seconds, and the remaining moisture was removed with filter paper. The image was obtained with an acceleration voltage of 120 kV using EF-TEM (Carl Zeiss Inc., Oberkochen, Germany).

### 2.4. Doxorubicin Delivery to U-87 MG: In Vivo Experiment

Thirty Balb/c female nude mice were obtained from Central Laboratory Animal Inc. (Seoul, Republic of Korea), housed under specific pathogen-free conditions, and handled in accordance with guidelines issued by the Yonsei University Institutional Animal Care and Use Committee (IACUC: YWCI-202206-006-04).

**Figure 1:**
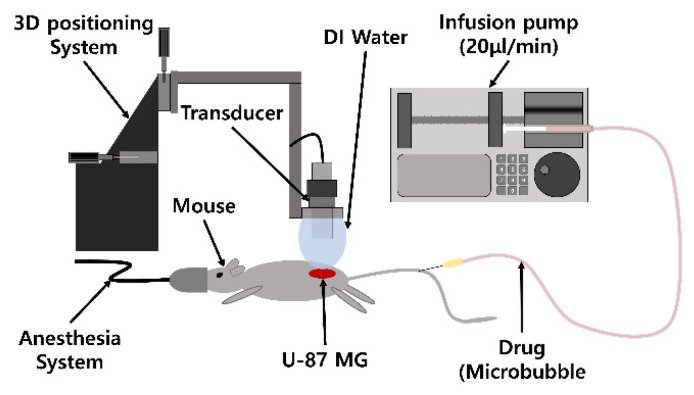
Animal experiment setup.

**Figure 2:**
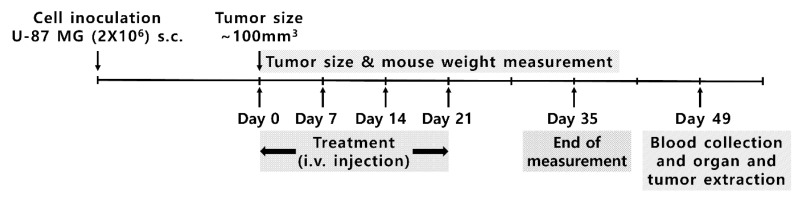
Animal experiment timeline.

The U-87 MG cell line was cultured in MEM supplemented with 10% FBS, penicillin (100 U/mL), and streptomycin (100 μg/mL). The U-87 MG cells (2 × 10^6^) were suspended in 100 μL of PBS and injected subcutaneously into the thigh of the right hind leg of the Balb/c mice at seven weeks of age. If the tumor volume reached 100 mm^3^, five comparative treatment experiments were conducted in vivo under the following conditions: (1) no treatment group as a base control; (2) 100 µL of diluted doxorubicin solution injected with an infusion pump (LSP02-1B; Longer Precision Pump Co. Ltd., Baoding City, China) at 20 µL/min for 5 min; (3) 100 µL of diluted doxorubicin solution and hollow echogenic liposomes (1.83 × 10^8^ expected bubbles) injected at 20 µL/min for 5 min with ultrasound at a pressure of 3.05 W/cm^2^ I_SPTA_ and a 10% duty cycle at 0.98 MHz at approximately 1 cm from the skin surface with a half-inch transducer; (4) 100 µL of diluted solution of doxorubicin-loaded echogenic liposomes injected at 20 µL/min for 5 min; and (5) 100 µL of diluted solution of doxorubicin-loaded echogenic liposomes injected at 20 µL/min for 5 min with ultrasound. Mice for each group were chosen randomly to avoid any possible systematic error. The experimental setup is depicted in Figure 1. The amount of doxorubicin was set to 3 µg/g according to body weight in all cases of doxorubicin use. In the case of echogenic liposomes, approximately 1.83 × 10^8^ echogenic liposomes were expected to be included according to particle count results. Each treatment experiment was conducted on four subjects every week, and tumor growth was observed for 35 days, as indicated in Figure 2. Upon the completion of treatment, all subjects were monitored for an additional two weeks.

Tumor size was measured with digital calipers, and tumor volume was estimated according to Equation (1) [38]. Upon the completion of the experiments, the mice were euthanized by cervical dislocation [39]. Blood and organs were collected for toxicity tests. A blood serum test was conducted on all subjects to investigate differences in toxicity according to the method used to deliver the doxorubicin. The levels of aspartate aminotransferase (AST), alanine aminotransferase (ALT), albumin, blood urea nitrogen (BUN), and lactate dehydrogenase (LDH) were measured quantitively with an AU480 analyzer (Beckman Coulter, CA, USA). For the statistical analysis, two outliers from each group were excluded.

(1) $$Tumor\;volume\;\left(mm^{3}\right)=\frac{1}{2}\times length\;\left(mm\right)\times width^{2}\;\left(mm^{2}\right)$$

## 3. Results

### 3.1. Population Characteristics of the Formed Echogenic Liposomes

**Figure 3:**
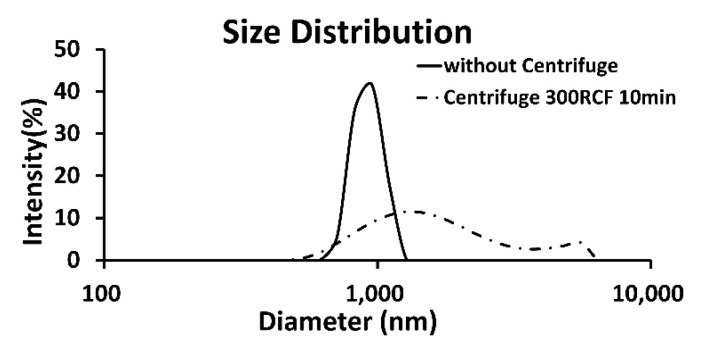
The size distribution changes after simple centrifugation. One milliliter of synthesized echogenic solution was centrifuged for 10 min with 300 RCF; then, the solution was remixed with gentle pipetting for ten times. The overall size distribution widened due to ten minutes of centrifugation.

The average population of particles reduced from 4.36×10^9^ particle/mL to 1.38×10^9^ particle/mL with a *p*-value of approximately 0.0001. The results indicate that a simple centrifugation reduced the bubble population significantly from the expected 50% to 30%. Additionally, a significant change in synthesized bubble distribution after centrifugation was easily observable in DLS analysis (Zetasizer Nano ZS90, Malvern Panalytical, Malvern, UK), as seen in Figure 3. Considering these factors, an additional purification process was decided to not be adopted in order to avoid significant changes in bubble characteristics. 

### 3.2. Visualization of Drug Load Ability Through Electron Microscopy

**Figure 4:**
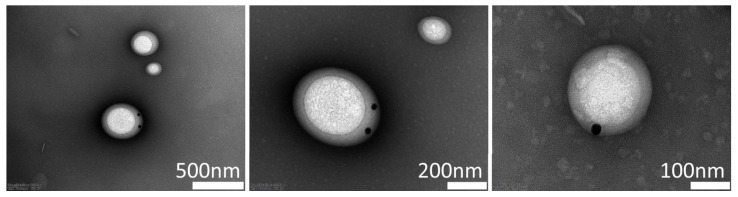
Transmission electron microscopy (TEM) images of synthesized echogenic liposomes with gold nanoparticles. After the synthesis of gold nanoparticle-loaded echogenic liposomes, uranyl acetate was stained for TEM as described in [32].

The gold nanoparticles appeared to aggregate and prevent the formation of echogenic liposomes during high-shear mixing. However, dozens of echogenic liposomes were successfully captured by transmission electron microscopy each time. As shown in Figure 4, some gold nanoparticles appeared to reside inside the liquid volume of echogenic liposomes. Given that no nanoparticles were overlapping the gas core area, they were most likely to be encapsulated inside of the liquid core instead of attaching on the surface.

### 3.3. Doxorubicin Delivery to U-87 MG: In Vivo Experiment in a Xenograft Model

**Figure 5:**
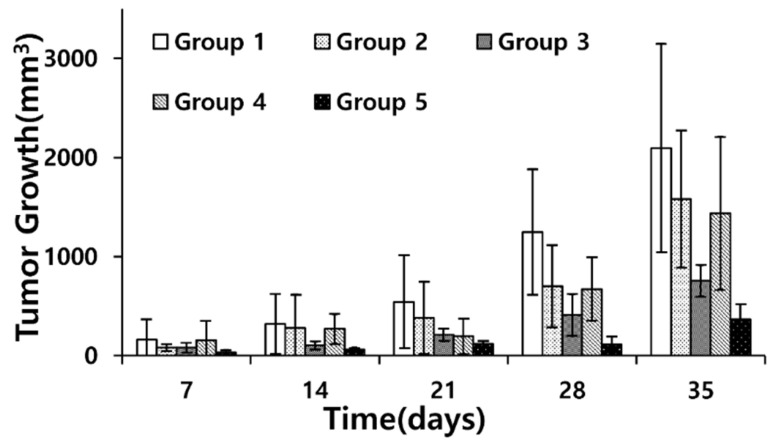
Tumor volume growth measurement (*n* = 4). Tumor volumes were measured once a week, and the net growths were calculated and visualized. Group 1: no treatment; group 2: doxorubicin; group 3: sonoporation with hollow microbubbles and doxorubicin mixture; group 4: doxorubicin-loaded echogenic liposomes without sonication; group 5: sonoporation with doxorubicin-loaded echogenic liposomes. Error bar indicates standard deviation.

The tumor volume measurements are shown in Figure 5. The tumor volume in each group was gradually increased to evaluate the effectiveness of each delivery method. Among these groups, group 5 (sonoporation with doxorubicin-encapsulated echogenic liposomes) showed the smallest increase in cancer volume. Compared with group 2 (simple administration of doxorubicin), tumor volume was approximately 25% on average. Performing ANOVA on the results from 35 days among groups 2–4 resulted in an F-value of 4.68 (*p*-value 0.02), and the following multiple comparison results showed that group 5 was clearly separated from group 2, as seen in Table 1. Although the statistical separation between groups 4 and 5 was statistically less significant, considering the *p*-value of 0.06 from large variation, sonoporation with doxorubicin-loaded echogenic liposomes shows the most efficient delivery with the smallest variation, which indicates that group 5’s treatment resulted in a consistently reliable delivery effect among all cases.

**Figure 6:**
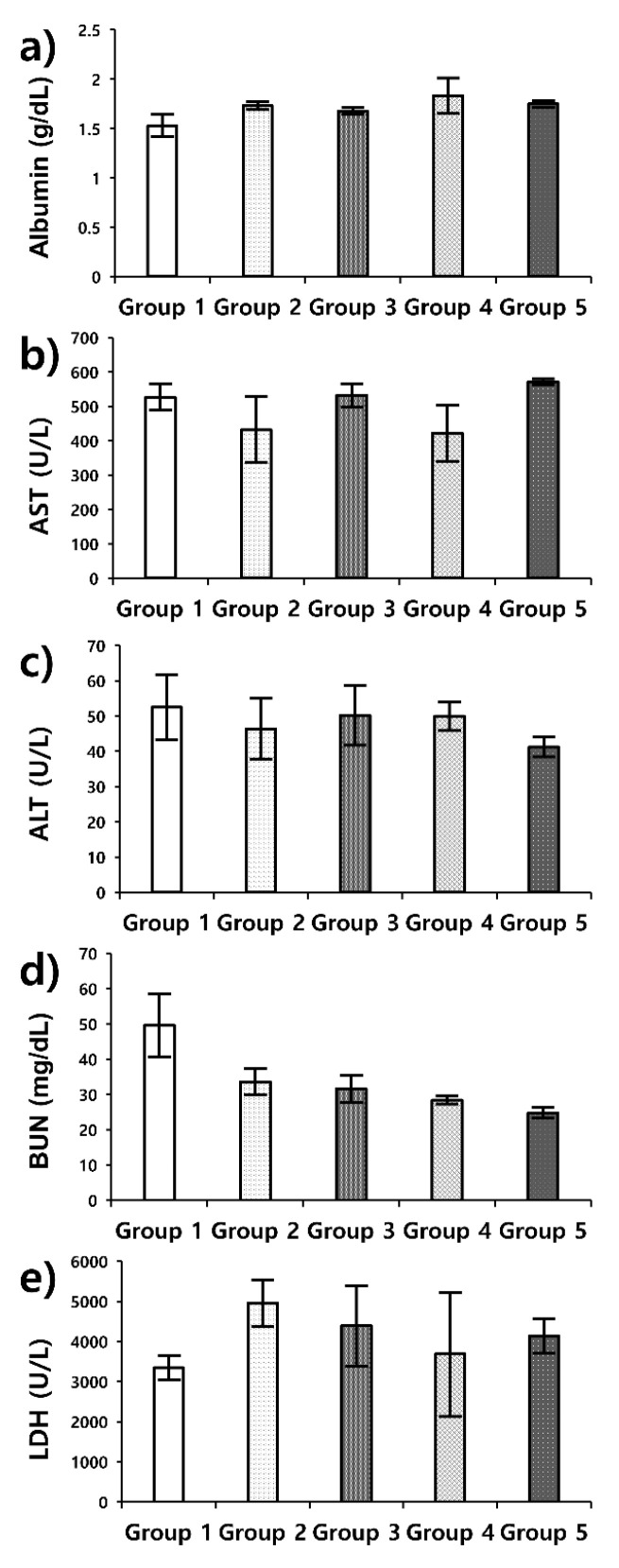
Blood serum analysis results. Blood was drawn 49 days after starting treatment. Results are show as mean, error bar indicates standard deviation; (**a**) albumin, (**b**) aspartate aminotransferase, (**c**) alanine aminotransferase, (**d**) blood urea nitrogen, and (**e**) low-density lipoprotein.

The blood serum analyses are summarized in Figure 6. Most markers show damage to organs, including the liver, kidney, and muscles, due to U87-MG cells. Even the treated subjects showed increases in AST, ALT, LDH, and BUN levels and a slight drop in albumin level compared with normal ranges (Table 2) [40]. According to previous reports [41,42,43], all of the markers were within the reasonable range of xenograft mouse models. The BUN values in group 5 were relatively low compared with the other groups and were in the normal range; any kidney damage was relatively minor. The overall treatment effect for group 5 appeared to be the strongest among the evaluated methods, according to blood serum tests.

## 4. Discussion

**Figure 7:**
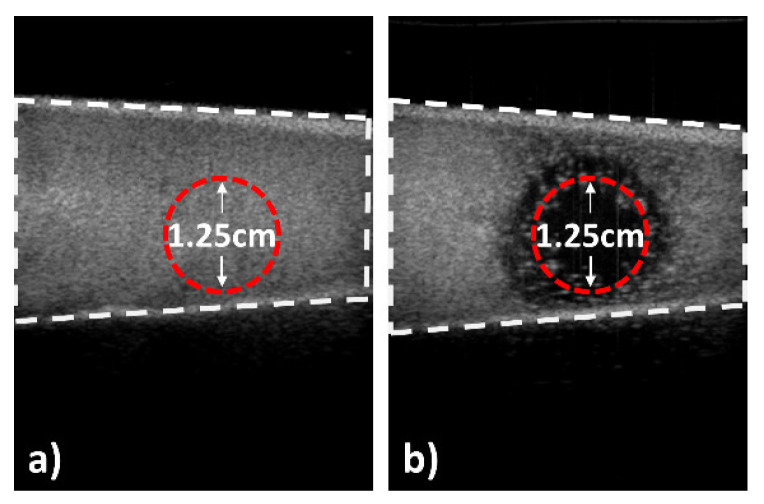
Bubble deletion in ultrasound imaging. An ultrasound system (PICO, Medison, Republic of Korea) with 5–9 MHz linear probe was used for observation. The frame rate was 20 frame/s, and the mechanical index (MI) was 0.9. The mixed solution of degassed water and echogenic liposome with a ratio of 100:1 was prepared and placed inside of the latex chamber. Ultrasound images before and after sonication were obtained. Ultrasound parameters were 1 MPa peak positive pressure with a 1% duty factor, 3.05 × 10^−1^ W/cm^2^ I_SPTA_. (**a**) is an ultrasound image before sonication, and (**b**) is an image right after starting sonication. The time between the two images was less than 100 ms. The affected area became dark immediately due to the destruction of echogenic liposomes. The dotted line indicates the active area of transducer which has similar size of darken area in the image. This indicates that the chosen parameters can be used for the effective disruption of echogenic liposomes over the sonicated area with a size that is close to the transducer, even though the focus size of the 6 dB transducer was only approximately 4 mm on the focal plane.

A large number of cavitation events under the ultrasound parameters of 1 MPa with a 1% duty factor was confirmed in the ultrasound images. If the echogenic liposome solution is mixed with 36 °C degassed water chamber at a ratio of 1:100, numerous bright spots can be seen in the ultrasound image (see Figure 7a). Whenever the ultrasound was sonicated with a transducer using the above-mentioned parameters, massive bubble deletion in the chamber was immediately observed, as shown in Figure 7b. Even though the intensity was 3.05 × 10^−1^ W/cm^2^ I_SPTA_ and the frequency of 1 MHz seemed low compared to the expected resonance frequency of the synthesized echogenic liposomes (~10 MHz range considering the size of gas core size), these chosen parameters ensured the disruption of echogenic liposomes.

The results from the in vivo experiments showed that sonoporation with doxorubicin-loaded echogenic liposomes showed statistically meaningful improved treatment effects with much smaller variance. The results imply that the proposed cavitation nuclei can be carriers of drug molecules for sonoporation and are worthy of further research for various applications, including the treatment of brain cancer. Although these results indicate the possible application of drug-loaded echogenic liposomes for cancer treatment, this does not mean that the research is at the final stage. Cancerous cells are not necessarily located next to blood vessels but can be located behind the cell–matrix or other cells. In the current xenograft model, U87-MG cells grew rapidly, and the formed blood vessel were not complete, allowing for loose tight junctions. This may lead to the enhanced permeability and retention (EPR) effect for drug delivery support [44]. To apply the proposed echogenic liposome in real cases, strategic approaches will be required such as securing the path through the BBB to cancerous cells with hollow bubbles and applying sonoporation with drug-loaded echogenic liposomes.

The tumor growth results might seem slightly contradictory to our previous in vivo experiments [45]. The addition of microbubbles during sonoporation could statistically significantly increase the effectiveness of drug delivery, while sonoporation combined with the mixture of hollow echogenic liposomes and doxorubicin was mildly improved in the current in vivo results. The reason could be related to the structure of the formed bubbles. As shown in a previous article [32], approximately 70 % of the formed bubbles are echogenic liposomes with a much smaller gas core compared to the equivalent simple microbubbles. Considering the cavitation event can lead to effective drug delivery only at the immediate vicinity of the gas–liquid barrier, echogenic liposomes may transport more internal liquid than external materials. Hollow echogenic liposomes have much lower doxorubicin concentrations within the internal liquid core compared to outside of them when a simple mixture of echogenic liposomes and doxorubicin is used. Logically, this could reduce the sonoporation effect compared to the usage of a simple microbubble mixture.

The limit of adopting a high-shear mixing method for synthesizing echogenic liposomes is the impurity of the formed bubbles. As shown in a previous report [32], approximately 70 % of the formed bubbles could be visually confirmed as echogenic liposomes while the remaining 30% were mostly simple bubbles with occasional simple liposomes. The difficulty of isolating echogenic liposomes is related to the relatively short half-life of around ten minutes. Considering these factors, the additional purification process was not adopted in this study. Accordingly, the results in this experiment include a certain level of uncertainty due to approximately 30% comprising simple microbubbles and occasional simple liposomes.

Liposomes themselves can be drug carriers. Generally, liposomes increase circulation times within the blood steam and help to transport encapsulated drug molecules into the cell membrane [46,47]. However, the efficacy of drug delivery of simple liposomes is generally not significant enough for practical use. Hence, an additional tunable method of liposome structures has been studied in depth [48]. Since the introduced simple liposomes are not specialized in their surface function, they were ruled out as major drug carriers under the ultrasonic field, at least in this study.

The quantitative measurement of the amount of doxorubicin inside echogenic liposomes can confirm their applicability. However, the short lifetime of echogenic liposomes is the critical limit for isolation and measurements once they are exposed to ambient environments. Only indirect methods were used to confirm the upload of any molecules. Based on these experiments, a large payload can be contained in echogenic liposomes, which can be used to effectively treat U-87 MG as sonoporation cavitation seeds.

## 5. Conclusions

The applicability of echogenic liposomes was evaluated using in vivo models. The effectiveness of doxorubicin-loaded echogenic liposomes in sonoporation was verified via comparative experiments. Although further research is needed on the controlled release of payloads into the target area, the proposed echogenic liposome is expected to be of use in sonoporation as a carrier and cavitation nuclei for various drugs used to treat cancers, including brain cancer.

## Figures and Tables

**Table 1:** Multiple comparison results between the two chosen groups of in vivo models.

Group Pairs	*p*-Value
2–3	0.18
2–4	0.98 × 10^−3^
2–5	0.31 × 10^−1^
3–4	0.31
3–5	0.73
4–5	0.61 × 10^−1^

**Table 2:** Normal range of blood serum chemistry [40].

Marker	Normal Range
Albumin	2.7–4.9 g/dL
AST	46–221 U/L
ALT	22–133 U/L
BUN	2–71 mg/dL
LDH	140–280 U/L

## Data Availability

The authors elected to not share data.
